# Warming Country Houses

**Published:** 1871-03

**Authors:** 


### WARMING COUNTRY HOUSES.

One of the most important items in the preservation of the general health is being comfortably warm all the time, for then we would never take cold. There should be a room in every farmer's family which should be kept at a temperature of not under sixty-five degrees Fahrenheit from daylight until bed-time, all winter, by stove or furnace heat; stove heat is better, because it will bring up the heat more quickly. When the farmer comes in from his work, he is generally over-heated and tired, both conditions making him greatly more susceptible of taking cold; or if, on the other hand, he is very cold from having been riding or engaged in something which has not involved activity enough to keep him adequately warm, a well-heated room is exceedingly grateful, and gradually raises the temperature of the surface of the body to its natural condition.

Large stoves consume less fuel in proportion than small ones, and give out more heat, hence are more economical.

It is a common error in the country to have too small stoves, so as to economize space, and under the mistaken notion that they consume less fuel in proportion. A circular stove six feet high and about two in diameter, lined with fire-brick two feet high, will keep a large room more equably warm, and maintain a purer atmosphere, with a very much less amount of fuel, than our common stoves. Stoves of this shape, made of porcelain, are used in Germany and Russia, where wood is grown for fuel; and from personal observation we think that about half the amount of wood is consumed, giving a greater, better, and more comfortable heat than we have here. In farmers' houses an immense amount of heat is used in warming all out-doors. The longer a flue is, the stronger the draft; all flues should be built from the ground, thus securing a good draft, and also saving millions of property every year from being burned, which is the case when flues are built on floors up through the rafters and roof.

Two sitting rooms on the same floor and one or two chambers above may be adequately warmed by one stove thus. Let the stove stand in one room and let a pipe of good size be sent through the partition into the adjoining room, where it should expand into a large drum; from this drum the ordinary pipe should extend through the floor into the chamber above, with a drum there if needed. Only a moderate amount of heat is needed in a chamber; but that moderate amount is needed in winter time. There is no advantage in going to bed in a cold room, nor in sleeping in a cold room, nor in getting up and dressing in a cold room; persons may survive it, many have lost health by it; to have the chill taken off the air on going to bed, and when dressing, is comfortable and healthful. A room under forty-five degrees is a cold room for a sleeping apartment, and sleeping in an atmosphere in-doors lower than that is always hurtful, is always positively pernicious, for the simple reason, that such a temperature causes the carbonic acid gas of a sleeping apartment to condense and settle in the lower part of the room, where it is breathed into the lungs, with all its pernicious results. Sleeping in a room cooler than above named is especially dangerous to feeble and aged or invalid persons, as it tends to cause inflammation of the lungs. Persons may sleep out of doors with impunity when the temperature is many degrees lower; that is because the out-door air is pure, is full of life, full of oxygen, without any admixture of in-door poisons, hence gives a vigor of circulation which keeps the whole body warmed to its natural point, resisting cold and all diseased conditions.

